# Synthesis and Evaluation of 1,3 Di-Substituted Schiff, Mannich Bases and Spiro Isatin Derivatives

**DOI:** 10.4103/0975-1483.63164

**Published:** 2010

**Authors:** P Mondal, M Banerjee, S Jana, A Bose

**Affiliations:** *Department of Pharmaceutical Chemistry, Institute of Pharmacy and Technology, Salipur, Cuttack, India*; 1 *School of Pharmaceutical Sciences, Siksha ‘O’ Anusandhan (SOA) University, Bhubneswer, Orissa, India*

**Keywords:** Analgesic, antipyretic, anti-inflammatory, isatin, Spiro compounds

## Abstract

Schiff bases of isatin with aminothiazole, its N-mannich bases and Spiro isatin derivatives were synthesized. Their chemical structures were confirmed by Infrared, 1H-Nuclear Magnetic Resonance data and elemental analysis. Antimicrobial evaluation was performed by the agar diffusion method against four pathogenic bacteria and two pathogenic fungi. Anti-inflammatory activity was tested by carragenin-induced rat paw edema and compounds were evaluated for analgesic action by the acetic acid-induced writhing method; Compounds Aa, Ab and A5, A6 were found to be active against bacteria and fungi. The compounds A3, A6, Aa and Ab showed anti-inflammatory activity, having a percentage protection value of 34.69, 32.65, 38.77 and 36.73 as compared with that of indomethacin, with % protection of 46.93. Similarly, the compounds Aa, Ab and A6 showed analgesic activity, with % protection of 67.51, 64.78 and 49.81 as compared with the standard with % protection of 79.56.

## INTRODUCTION

Isatin (1-H indole 2-3 dione) are synthetically versatile substances that are employed for the synthesis of a large variety of heterocyclic compounds, including some drugs. Over the years, the molecules with isatin show diversified biological activities, e.g. antibacterial,[1] anticonvulsants,[2] antiviral[3] and antitubercular[4] activities. Schiff and mannich bases of isatin have shown good antimicrobial activities against gram-negative bacteria and fungi.[3] A number of Spiro compounds derived from isatin are important pharmacophores and exhibit promising biological activities,[5] such as analgesic, fungicidal and anti-inflammatory activity. Thiazole and its derivatives are reported to show good antibacterial, anti-inflammatory and analgesic[6] activity. Hence, it was thought worthwhile to synthesize new Schiff, mannich and Spiro isatin derivatives and investigate them for biological and pharmacological activities. The key starting materials, 1-H-indole 2-3 dione and 2-aminothiazole, were refluxed in the presence of alcohol and glacial acetic acid to get Schiff base 3 (1’-3’thiazole 2-yl imino) 1-3 dihydro 2-H-indole-2-one (A); mannich bases are prepared with different secondary amines in the presence of formaldehyde (A_1_ -A_6_). Meanwhile, Spiro isatin derivatives are prepared with Azetidine (A_a_) and thiazolidone (A_b_) in the presence of chloroacetyl chloride (Cl-CH_2_ -CO-Cl) and thioglycolic acid (SH-CH_2_ -COOH).

## MATERIALS AND METHODS

Melting points are recorded in open capillary tubes and are uncorrected. The IR spectra (KBr) were recorded on a SHIMADZU FTIR-8300, spectrophotometer, Japan. The H-NMR spectra were recorded on a Bruker Advance-400 MHz spectrometer, USA. Purity of the compounds was checked by Thin layer chromatography For analgesic, anti-inflammatory activity studies, adult healthy albino rats (Swiss strain) of either sex weighing 80–120 g were used. All the animals were maintained under standard conditions and had access to pelleted animal feed and water. The study protocol was approved by the Institutional Animal Ethics Committee.

### Synthesis[7] of 3(1’-3’Thiazole-2-yl Imino) 1-3 Dihydro-2H Indole-2-One (A)

Equimolar quantity of isatin and 2-amino thiazole was taken in a round bottom flask, dissolving the content with a sufficient amount of ethanol and two to three drops of glacial acetic acid was added. The content was refluxed for 4–5 h and then poured into ice cold water. The solid obtained was filtered, dried and recrystallized from ethanol.

### Synthesis[7] of 1[(Diphenyl Amino) Methyl]-3-(1’-3’ Thiazole-2-yl Imino) 1-3 Dihydro-2H Indole-2-One (A_1_)

To a slurry containing A (0.003 mol), ethanol (5 ml) and 37% formalin (1 ml), diphenylamine (0.003 mol) was added slowly with good stirring. The reaction mixture was cooled and allowed to stand at room temperature for 1 h, with occasional shaking. Then, it was warmed on a steam bath for 15 min, cooled and the product was recovered. The product was recrystallized from chloroform–methanol (1:1) mixture.

The compounds 1(piperazin-1-yl) methyl-3-(1’-3’ thiazole-2-yl imino) 1-3 dihydro-2H indole-2-one (A_2_), 1[(dicyclohex-yl) methyl]-3-(1’-3’ thiazole-2-yl imino) 1-3 dihydro-2H indole-2 one (A_3_), 1[(dimethylamino) methyl]-3-(1’-3’ thiazole-2-yl imino) 1-3 dihydro-2H indole-2 one (A_4_), 1-[(diethylamino) methyl]-3-(1’-3’ thiazole-2-yl imino) 1-3 dihydro-2H indole-2 one (A_5_) and 1-[(morpholin-4“yl) methyl]-3-(1’-3’ thiazole-2-yl imino) 1-3 dihydro-2H indole-2 one (A_6_) were prepared by following a similar procedure.

### Synthesis of 3’Chloro-1-(1”-3”Thiazo-2-yl) 4’-H-Spiro [Azetidine-2, 3’Indole] 2’4-(1-H) Di One (A_a_)[8]

Compound 3(1’-3’thiazole-2-yl imino) 1-3dihydro-2H indole-2-one (A) (0.01mol) was dissolved in 1-4 dioxan (20 ml) in a 100 ml beaker and trimethyl amine (0.001 mol) was added to it. Then, the contents of the beaker were stirred and chloroacetyl chloride (0.001 mol) was added slowly, continuing stirring of contents of the beaker for 8 h. The resulting solution was poured into ice cold water, filtered, dried, recrystallized from chloroform–methanol (1:1) and added in a round bottom flask. The content of the flask was refluxed for 12–14 h, the product was recovered in ice cold water, filtered, dried and finally recrystallized from chloroform–methanol (1:1) mixture.

### Synthesis of 3’Chloro-1-(1”-3”Thiazo-2-yl) 4’-H-Spiro [Indole-3,2-(1,3) Thiazolidine] 2’4-(1-H) Di One (A_B_)[5]

Equimolar quantity of compound A and thioglycollic acid was dissolved in a round bottom flask. Then, 1,4-dioxane and a pinch of zinc chloride was added to it. The contents of the mixture were refluxed for 12–14 hr, concentrating the content of the flask, and poured into ice cold water. The solid mass was collected and dried. The product was recrystallized from chloroform–methanol mixture (1:1).

### Antimicrobial activity

The *in vitro* antimicrobial activity[9] was carried out against 24-h-old cultures of microorganisms by the cup–plate method. Compound A_1_ -A_6_and A_a_, A_b_were tested against four pathogenic bacteria, *Pseudomonas mirebelis, Pseudomonas auroginosa, Escherichia* coli and *Staphylococcus aureus*. The antifungal activity was tested against *Aspergillus niger* and *Candida albicans*. Ampicillin and cotrimoxazole was taken as an internal standard for antibacterial and antifungal activity, respectively. The compound was tested at a concentration of 100 µ/ml in DMF against all organisms. The zone of inhibition was compared with the standard drug after 24-h incubation at 37°C for antibacterial and 48 h at 25°C for antifungal activity.

### Analgesic activity

The analgesic activity[10] was evaluated by the acetic acid-induced writhing test[6] using adult Swiss albino mice of either sex. In this method, mice are made to writhe by a simple intraperitonial injection of 0.6% v/v aqueous acetic acid (0.1 ml/kg). Test substances were administered 30 min before the injection of acetic acid. The number of writhes (full extension of hind paws) was recorded.

### Anti-inflammatory activity

The anti-inflammatory activity[10] was evaluated by rat paw edema. Edema represents the early phase of inflammation and carragenin-induced paw edema is the simplest and most widely used method[8] for studying the anti-inflammatory activity of the chemical compounds. This method is based on the plethysmographic measurement of carragenin-induced acute rat paw edema. For this study, Wister rats of either sex, weighing between 80 and 100 g were divided into 10 groups of five animals each. Group1 serves as a solvent control, and received 0.5% Carboxy Methyl Cellulose in normal saline orally. Group 2 received indomethacin (10 mg/kg) in solvent as a standard. Groups 3–10 received test drugs at a dose of 100 mg/kg orally. These drugs were administered 1 h before the injection of an irritant, carragenin. After 1 h, all the animals were injected subcutaneously with a suspension of carragenin in CMC solution (0.1 ml) to the left hind paw in the subplantar region and the paw volume was measured immediately. After 3 h, the paw volume was measured for the control, standard and test groups. Percentage inhibition of paw volume was calculated.

## RESULTS AND DISCUSSION

The structures of newly synthesized compounds were elucidated by IR, ^1^H-NMR and elemental analysis and are reported as follows:

The IR spectrum of **A** in KBr (in cm^-1^): 3446 (NH str), 1728 (C=O), 1610.95 (C=N), 1469.01 (CH=CH of Ar), 1096.66 (C-S-C).

^1^H-NMR of **A** (δ values):11.04 (S, 1H, NH), 6.89–7.61 (m, Ar-H), 3.39 (1H,CH), 3.16 (1H, CH).

IR of **A_1_**(in cm^-1^): 2890 (CH_2_), 1730 (C=O), 1670 (C=N), 1459.37 (CH=CH), 1200 (C-N), 1090 (C-S-C), 766.888 (C=C aromatic hydrocarbon).

^1^H-NMR of A_1_ (δ values): 6.89–7.09 (m, Ar-H), 2.50 (S.2H, N-CH_2_), 3.38 (S, 2H, CH of thiazole).

IR of **A_2_**(in cm^-1^): 2832 (CH_2_), 1720 (C=O), 1609 (C=N), 1480 (CH=CH), 1363 (C-N), 1164.30 (C-S-C str), 3305 (N-H).

^1^H-NMR of **A**_2_ (δ values): 6.89–7.92 (m, Ar-H), 4.38 (S, 1H, N-H), 3.06–3.38 (δ, 1H, CH), 1.23 (t, 2H, CH_2_piperazine).

IR of **A_3_**(in cm^-1^): 2946 (CH), 2854 (C-H), 1718.84 (C=O), 1647.58 (C-N), 1517 (C=N), 1458 (CH=CH of Ar), 1105 (C-S-C).

IR of **A_5_**(in cm^-1^): 2942 (CH_2_), 1735 (C=O), 1676 (C=N), 1654 (C-N), 1542 (CH=CH of Ar), 1042 (C-S-C str), 758 (C=C).

IR of **A_6_**(in cm^-1^): 2396 (CH_2_str), 1718 (C=O), 1560 (C-N str), 1654 (C=N), 1542 (CH=CH of Ar), 1108 (C-S-C str), 1005 (C-O-C str), 717 (C=C Ar).

IR of **A_a_**(in cm^-1^): 3445 (NH str), 1720 (C=O), 1710 (C=O), 1640 (C-N), 649 (C-Cl).

^1^H-NMR **A_a_**: 11.08 (s, 1H, NH), 6.93–7.79 (m-Ar-H), 3.39 (δ,1H, CH of thiazole), 2.27 (s, 1H, CH, CH_2_Cl).

IR of A_b_(in cm^-1^): 3442 (NH str), 1724 (C=O), 1708 (C=O), 1645 (C-N), 1090 (C-S-C).

^1^H-NMR of **A_b_** (δ values): 11.08 (s, 1H, NH), 6.71–7.78 (m-Ar-H), 3.39 (δ, 1H, CH of thiazole), 2.50 (s, 2H, CH_2_).

The characterization of synthesized compounds is given in Table 1. Analytical data of the compounds supports the proposed structures.

**Table 1:** Characterization data of the synthesized compounds

Compounds	M.P (°C)	Yield%	Molecular formula	Found (calculated) %		
				C	H	N
A_1_	108–110	59	C_24_H_18_N_4_OS	58.52 (58.72)	5.03 (5.23)	21.12 (21.39)
A_2_	238–240	68	C_16_H_17_N_5_OS	69.90 (70.22)	4.21 (4.41)	13.40 (13.65)
A_3_	110–114	72	C_24_H_30_N_4_OS	67.01 (68.21)	6.89 (7.16)	12.89 (13.28)
A_4_	220–223	72	C_14_H_14_H_4_OS	58.60 (58.72)	4.81 (4.93)	19.40 (19.57)
A_5_	252–254	61	C_16_H_18_N_4_OS	61.01 (61.12)	5.72 (5.77)	17.81 (17.82)
A_6_	248–250	65	C_16_H_16_N_4_O_2_S	58.71 (58.82)	4.80 (4.91)	16.99 (17.06)
A_a_	343–344	65	C_13_H_8_N_3_SO_2_Cl	50.88 (51.47)	2.47 (2.64)	13.53 (13.74)
A_b_	340–342	62	C_13_H_9_N_3_S_2_O	51.12 (51.47)	2.83 (2.99)	3.63 (13.85)

Results of antimicrobial activity, analgesic activity and anti-inflammatory activity are shown in Tables 2–4, respectively. The results of the antimicrobial activity revealed that the compounds A_3_, A_5_, A_6_, A_a_and A_b_ were found to be active against bacteria and fungi. The compounds A_3_, A_6_, A_a_ and A_b_ showed significant analgesic activity, with percentage of inhibition of 43.69, 49.81, 67.51, 64.78, respectively, as compared to indomethacin, with percentage inhibition of 79.56. The compounds A_a_, A_6_, A_b_, showed considerable anti-inflammatory activity, having percentage protection of 38.77, 32.65 and 36.73 as compared with indomethacin, having percentage protection value of 46.93.

Thus, it is observed that the synthesized compounds showed promising antimicrobial, analgesic and anti-inflammatory activities. Further studies are needed to discover new novel compounds of this class with profound activities.

**Table 2:** Results of antimicrobial activity

Compounds	Zone of inhibition* (mm)					
	Antibacterial activity			Antifungal activity		
	*P. mirebelis*	*P. auroginosa*	*E. coli*	*S. aureus*	*A. niger*	*C. albicans*
A_1_	12	14.2	10	9.5	22.2	17.3
A_2_	13	12.9	10	8.3	21	16.5
A_3_	10	10.3	11.2	9.8	23	20
A_4_	11.1	11	10	8.8	17	17.8
A_5_	11	11.2	13.6	9.3	21	15.3
A_6_	12.2	12.5	14	9.1	22.9	19
A_a_	15.2	17	20	10	5	21.2
A_b_	13.5	16.1	17.5	10	24.3	20
Ampicillin	23	22.5	24	26	27.6	28
Cotrimox zoleDMF	-		-	-	-	-

* average of three readings.

**Table 3:** Analgesic activity of the compounds

Compound code	Dose (mg/kg)	Number of writhing movements	% of protection
0.5% CMC	-	58.8 ± 0.95	-79.5632.1128.46
Indomethacin	10	11.2 ± 4.32**	43.7933.2127.91
A_1_	100	37.2 ± 3.21*	49.8167.5164.78
A_2_	100	39.2 ± 3.48	
A_3_	100	30.8 ± 2.23*	
A_4_	100	36.6 ± 2.97*	
A_5_	100	39.5 ± 3.27	
A_6_	100	27.5±2.85**	
A_a_	100	17.8 ± 2.19**	
A_b_	100	19.3 ± 2.98**	

Values are expressed as mean ± SE (*n* = 6).

* *P*<0.05,

** *P*<0.01 and ***P<0.001 compared with vehicle control (ANOVA followed by Dunnet’s *t*-test).

**Table 4:** Anti-inflammatory activity of the compounds

Compound code	Group	Volume of mercury displaced (ml)		
		1 h (% of protection)	2 h (% of protection)	3 h (% of protection)
0.5% CMC	1	0.49 ± 0.013	0.55 ± 0.033	0.59 ± 0.022
Indomethacin	2	0.26 ± 0.012 (46.93)	0.37 ± 0.022 (32.72)	0.39 ± 0.012 (33.89)
A_1_	3	0.41 ± 0.025* (16.32)	0.54 ± 0.029 (1.81)	0.45 ± 0.022** (23.72)
A_2_	4	0.48 ± 0.041 (2.02)	0.54 ± 0.012 (1.81)	0.57 ± 0.032 (3.38)
A_3_	5	0.32 ± 0.041** (34.69)	0.53 ± 0.034 (3.36)	0.46±0.030** (22.03)
A_4_	6	0.47 ± 0.033 (4.08)	0.52 ± 0.036 (5.54)	0.43±0.032** (27.11)
A_5_	7	0.46 ± 0.043 (6.12)	0.54 ± 0.012 (1.81)	0.56 ± 0.032 (1.69)
A_6_	8	0.33 ± 0.032** (32.65)	0.39±0.031* (29.09)	0.46±0.016** (22.03)
A_a_	9	0.30 ± 0.043** (38.77)	0.37±0.013** (32.72)	0.45±0.041*** (23.72)
A_b_	10	0.31 ± 0.041** (36.73)	0.38±0.033** (30.90)	0.46±0.061** (22.03)

Values are expressed as mean ± SE (n = 6).

* *P*<0.05

** *P*<0.01

*** *P*<0.001 compared with vehicle control (ANOVA followed by Dunnet’s *t*-test).

## References

[1] Verma RS, Nobles WL (1975). Antiviral antibacterial and antifungal activities of Isatin N-mannich bases. J Pharm Sci.

[2] Popp FD, Parson R, Donigan BE (1980). Synthesis of potential anticonvulsants: condensation of Isatin with acetone and related ketones. J Pharm Sci.

[3] Pandeya SN, Sriram D, Nath G, DeClercq E (1999). Synthesis, antibacterial, antifungal and anti HIV activities of Schiff and mannich bases derived from Isatin derivatives and N-[4-(4’chlorophenyl) thiazol-2-yl] thiosemicarbazide. Eur J Pharm Sci.

[4] Tran VH, Nguyen QD, Le NV, Le TT (2000). Study on antituberculosis effect of some thiosemicarbazones and isonicotinyl hydrazone derivatives of Isatin and 5-halogino Isatin. Tap Chi Douc Hoc.

[5] Mashelkar CU, Rane MD (2005). Synthesis of some Isatin based novel heterocycles and their biological activity studies. Indian J Chem (Sec-B).

[6] Venugopala KN, Waheed A, Khan SA (2001). Synthesis of carboxamide of 2 amino-4’-(6-bromo-3 coumarinyl) thiazole as analgesic and anti-inflammatory agent. Indian J Heterocyclic Chem.

[7] (2002). Synthesis antibacterial and antifungal activities of N-mannich bases of 3[N^2^ pyrimethaminyl imino] isatin. Indian J Pharm Sci.

[8] Mistry K, Desai KR (2005). Synthesis and antibacterial activities of spiroazetidine and pyrazoleimines. Indian J Chem (Sec B).

[9] Purohit SS (2002). Microbiology fundamentals and applications.

[10] Ghosh MN (1984). Fundamentals of experimental pharmacology.

